# Evaluating borrowers’ default risk with a spatial probit model reflecting the distance in their relational network

**DOI:** 10.1371/journal.pone.0261737

**Published:** 2021-12-31

**Authors:** Jong Wook Lee, So Young Sohn

**Affiliations:** Department of Information and Industrial Engineering, Yonsei University, Seoul, Republic of Korea; Politecnico di Torino, ITALY

## Abstract

Potential relationship among loan applicants can provide valuable information for evaluating default risk. However, most of the existing credit scoring models either ignore this relationship or consider a simple connection information. This study assesses the applicants’ relation in terms of their distance estimated based on their characteristics. This information is then utilized in a proposed spatial probit model to reflect the different degree of borrowers’ relation on the default prediction of loan applicant. We apply this method to peer-to-peer Lending Club Loan data. Empirical results show that the consideration of information on the spatial autocorrelation among loan applicants can provide high predictive power for defaults.

## 1. Introduction

Credit risk management is very important for service firms in the lending business. To predict the probability of default of loan applicant that is essential for credit risk management, machine learning models use two types of borrower information: standard “hard” information and nonstandard “soft” information [1]. The former directly reflects the loan applicants’ financial status or creditworthiness, while the latter includes those that do not have a direct relationship to the credit applicant’s financial status or creditworthiness such as age or residence. Existing studies have shown that not only hard information but also soft information, which is less relevant to their financial condition, is helpful in predicting default risk [1–5]. While both hard and soft information has been used in most credit scoring models, what is missing is the potential relation among loan applicants. Relationship among loan applicants that are at high risk of default can also provide valuable information for evaluating default risk [6–8].

In this study, we use a borrower relationship network based on the borrowers’ information provided for loan applications. This network is utilized as a spatial weight matrix for a spatial probit model that reflects different degrees of borrowers’ relation for the prediction of a loan default. Our proposed approach is applied to peer-to-peer (P2P) lending.

Online P2P lending allows individuals to lend money to other individuals through online platforms without the intervention of a financial institution. These online P2P lending platforms are gaining popularity due to their low operating costs compared with traditional lending programs [9]. However, online P2P lending faces a significant problem, such as information asymmetry between borrowers and lenders, that is, the reliability of a borrower’s credit is unknown to the lender [10]. Therefore, the use of relationship information among borrowers beyond those provided on the P2P platform is necessary. As it is difficult to discover realistic relationship information between borrowers in a P2P landing platform, this study defines the data-driven latent relationships between borrowers in terms of the similarity of their hard and soft information. We expect that the data-driven latent relationships information between borrowers can improve default risk prediction.

This paper is organized as follows. Section 2 reviews prior studies on default prediction in online P2P lending. Section 3 explains the methodologies employed, and Section 4 explores the Lending Club Loan (LCL) dataset used for this study. Finally, Section 5 presents the results, and Section 6 discusses the results, limitations, and suggestions for improvement.

## 2. Literature review

Models for default risk prediction in P2P lending services are divided into three categories: the probability of default (PD), exposure at default (EAD), and loss given default (LGD). Among them, PD models have been explored steadily [11]. The PD model predicts borrower’s default using classification models based on the statistical or machine learning approaches. Statistical methods have the advantage of being able to quantitatively show the effect of each factor on the borrowers’ default [12]. Emekter et al. [13] used a logistic regression model to predict the default probability of borrowers and found that Fair, Isaac and Company scores are a very important factor. However, statistical methods have the disadvantage of requiring strong assumptions in the observed data [14]. Meanwhile, machine learning methods have strong default prediction performance without requiring any statistical assumptions. These models include neural network [15], support vector machine [16, 17], and random forest [18]. However, these models have a fatal drawback, that is, individual factors do not directly show the effect on borrowers’ default.

It is also important to choose the optimal features used to predict default risk. Generally, hard information can reflect borrowers’ repayment ability [19], while soft information can reflect borrowers’ repayment willingness [20]. Hard information plays an important role in explaining default risk because it directly represents the borrowers’ financial status. However, online P2P lending platforms have difficulty collecting sufficient hard information. To overcome these limitations, the importance of soft information that is not related to the borrowers’ financial status is increasingly emphasized. Lin et al. [21] discovered that information on gender, age, educational level, and marital status play a significant role in predicting default. Recently, unstructured data, such as text and image information, as well as structured data, have been used as soft information. Dorfleitner et al. [22] used textual soft information containing a description of the loan purpose such as text length, spelling errors, and the presence of positive emotion-evoking keywords. Jiang et al. [23] used a topic model to extract representative features from descriptive text concerning loans.

However, few studies have used information on the relationship among individual borrowers in online P2P lending services. Calabrese et al. [24] defined bank networks by estimating interbank relationships as aggregate claims to predict bank contagion. Agosto et al. [6] defined business networks by estimating inter-company relationships as aggregate trade volumes to predict business default from P2P platforms that specialize in business lending. Unlike for banks and companies, obtaining quantitative indicators of relationships among individuals is difficult. In this study, we propose a network definition among individual borrowers and use this relationship information as independent information.

## 3. Methodology

### 3.1 Spatial probit model

Generally, the latent response model is the method used to fit the binary response variable Y as a regression model [25]. The model used in this study is a spatial probit model, which has a spatial autoregressive structure and can be used with a binary response variable. Taking the latent underlying quantity as being represented by a continuous variable $Y_{i}^{*}$, we consider the observation mechanism as

$$Y_{i}=\{\begin{array}{ll} 1, & Y_{i}^{*}>0\\ 0, & \text{otherwise} \end{array}$$ (1)

with i = 1, 2, ⋯, n where n is the number of observations. We implement the spatial structure with an autoregressive model specification, such that

$$Y^{*}=\rho WY^{*}+X\beta +\varepsilon ,$$ (2)

where Y* is a continuous latent vector; **X** represents an n × k matrix of explanatory variables with related coefficient vector **β**; **W** is a spatial lag weights matrix with **ρ** as the associated coefficient; and **ε** is the error term.

This spatial probit model implies heteroskedastic errors **e** as follows:

$$Y^{*}={\left(I-\rho W\right)}^{-1}(X\beta +\varepsilon )={\left(I-\rho W\right)}^{-1}X\beta +e$$ (3)

where **e** = (**I** − **ρW**)^−1^**ε** with variation: $var(e)=\sigma_{\varepsilon }^{2}{\left[{\left(I-\rho W\right)}^{\prime }(I-\rho W)\right]}^{-1}$.

Calabrese and Elkink [26] reviewed various methods for estimating parameters **ρ** and **β** in Eq (3). Among them we performed parameter estimation using the generalized method of moments (GMM) proposed by Pinkse and Slade [27], which derive the GMM equations from the likelihood function. This method is extended by Klier and McMillen [28] to the logit model. It is more robust than the maximum likelihood estimation because it does not depend on the assumption that the error term follows a normal distribution [27].

A GMM estimator is defined as follows:

$$\hat{\theta }=arg\;\underset{\theta }{\text{min}}\;u^{\prime }ZMZ^{\prime }u$$ (4)

where **θ** = [**ρ**, **β**], **u**_**i**_ **= y**_**i**_ **− p**_**i**_, $p_{i}=Pr[y_{i}=1]=\frac{exp({\left(I-\hat{\rho }W\right)}^{-1}X^{*}\hat{\beta })}{1+exp({\left(I-\hat{\rho }W\right)}^{-1}X^{*}\hat{\beta })},\;X_{i}^{*}=\frac{X_{i}}{\sigma_{i}}$; **σ**_**i**_ is a diagonal element of covariance matrix [(**I** − **ρW**)′(**I** − **ρW**)]^−1^; **Z** is a matrix of instruments; and **M** is a positive definite matrix that is generally initialized to an identity matrix. We define the instrument matrix **Z** = {**X**, **WX**, **W**^**2**^**X**, **W**^**3**^**X**}, as proposed by Kelijian and Prucha [29].

To estimate the parameter, **θ**, we use a two-step estimation procedure:

1. First, fix **ρ** = **ρ**_**0**_, then estimate the **β**_**0**_ with GMM and
2. Find the optimal value of $\hat{\theta }=[\hat{\rho },\hat{\beta }]$ through GMM as the initial value of **θ**_**0**_ = [**ρ**_**0**_, **β**_**0**_] found in (1).

The estimated spatial lag $\hat{\rho }$ is used to test the statistical significance of **ρ** by the Lagrange Multiplier (LM) test proposed by Anselin [30]. The LM statistic for spatial lag $\hat{\rho }$ is defined as:

$${LM}_{\rho }={\left[u^{\prime }Wy/(u^{\prime }u/n)\right]}^{2}/D$$ (5)

where $D=\left[\left(WX\beta \right)\prime \left(I-X{\left(X^{\prime }X\right)}^{-1}X^{\prime }\right)\left(WX\beta \right)/{\hat{\sigma }}^{2}\right]+tr\left(W^{2}+{\text{W}}^{\prime }\text{W}\right)$ with ${\hat{\sigma }}^{2}=[e_{0}-\hat{\rho }e_{L}]\prime [e_{0}-\hat{\rho }e_{L}]/n$, e_**0**_ **= y − X(X′X)**^**−1**^**X′y**, and **e**_**L**_ **= y − X(X′X)**^**−1**^**X′Wy**.

The spatial lag weights matrix between borrowers on the P2P platform, W, is defined in Section 3.2.

### 3.2 Borrowers`relation network

In this study, we construct a network with each borrower as a node and the distance between them as an edge to represent the relationship between the borrowers. The distance between them is defined as the degree of similarity in terms of their hard and soft information. Similarity between numeric information is easily defined by Euclidean distance, but defining similarity between categorical information is a challenge. We use a method proposed by Ahmad and Dey [31] to calculate the distance between borrowers with mixed numeric and categorical information.

Let us assume **B**_**i**_ and **B**_**j**_ are two borrowers with **m** hard and soft information attributes: **X**_**1**_, …, **X**_**m**_. The two borrowers may be represented as **B**_**i**_ = {**X**_**i1**_, **X**_**i2**_, …, **X**_**im**_} and **B**_**j**_ = {**X**_**j1**_, **X**_**j2**_, …, **X**_**jm**_} where the first **m**_**r**_ attributes are numeric, the next **m**_**c**_ attributes are categorical, and **m**_**r**_ + **m**_**c**_ = **m**. The distance between **B**_**i**_ and **B**_**j**_, denoted by **Dist**(**B**_**i**_, **B**_**j**_) is computed as follows:

$$Dist(B_{i},B_{j})=\sum_{t=1}^{m_{r}}{\left(s_{t}(X_{it}-X_{jt})\right)}^{2}+\sum_{t=m_{r}+1}^{m}{\left(\delta (X_{it},X_{jt})\right)}^{2}.$$ (6)

where **s**_**t**_ is the significance of the t-th numeric attribute, and **δ**(**X**_**it**_, **X**_**jt**_) is a distance function between the t-th categorical attributes in **B**_**i**_ and **B**_**j**_. The distance between two distinct values, **c**_**1**_ and **c**_**2**_, of any categorical attribute **X**_***t***_ is given by:

$$\delta (c_{1},c_{2})=(\frac{1}{m-1})\sum_{t`=\;1,\cdots ,m,\;t\neq t`}\delta^{tt`}(c_{1},c_{2})$$ (7)

where **δ^tt`^** (**c**_**1**_, **c**_**2**_) = **P**_**t**_(**c`** |**c**_**1**_) + **P**_**t**_(~**c`** |**c**_**2**_) − **1**, **c`** denotes a subset **C** of values of **X**_**t`**_ that maximizes the quantity **P**_**t**_(**c`** |**c**_**1**_) + **P**_**t**_(~**c`** |**c**_**2**_); ~**c`** denotes the complementary set of values occurring for attribute **X**_**t`**_; and **P**_**t**_(**c`** |**c**_**1**_) denotes the conditional probability that an element having value **c**_**1**_ for **X**_**t`**_ has a value belonging to **c`** for **X**_**t`**_. To compute the significance of normalized numeric attributes, we discretize them to have **L** equal intervals: **u**[**1**], **u**[**2**], ⋯, **u**[***l***]. The significance of the t-th numeric attribute, **s**_**t**_, is computed as:

$$s_{t}=\sum_{l_{1}=1}^{L-1}\sum_{l_{2}>l_{1}}^{L}\delta (u_{t}[l_{1}],u_{t}[l_{2}])/(L(L-1)/2).$$ (8)

The relationship between two borrowers (**B**_**i**_ and **B**_**j**_) is mapped so that the closer the distance is, the stronger the relationship. We use double-power distance weights, and the degree of relationship between **B**_**i**_ and **B**_**j**_ is evaluated as follows:

$$W_{ij}=\{\begin{array}{cc} {\left[1-{\left(\text{Dist}\left(B_{i},B_{j}\right)/d\right)}^{2}\right]}^{2}, & 0\leq \text{Dist}\left(B_{i},B_{j}\right)\leq d\\ 0, & \text{Dist}\left(B_{i},B_{j}\right)>d \end{array}$$ (9)

where **d** donates the maximum radius of influence (bandwidth). To use **W**_**ij**_ as a spatial weight matrix, row normalization is performed.

### 3.3 Evaluation metric

To measure the performance of the proposed spatial probit model, we used the following evaluation metrics: accuracy, precision, recall, F1 score, and area under the receiver operator characteristic (ROC) curve. These 4 indicators are the most used indicators for performance evaluation of binary classification tasks such as default prediction. The accuracy is the most intuitive performance indicator of a classification model and is defined as the ratio of correct to total predictions. The precision is the percentage of borrowers that actually defaulted out of those who were predicted to default. The recall is the percentage of borrowers predicted to default out of those actually defaulted. The F1 score is the harmonic mean of the precision and recall. Precision, recall, and f1 score are used as important indicators in a credit scoring task where borrowers with default is much less than borrowers with fully paid [32]. The ROC curve for a binary classification problem represents the true positive proportion as a function of the false positive proportion.

## 4. Data

We used LCL data from Lending Club, the largest online credit marketplace offering P2P lending worldwide. This data is open to public and provides 2.26 million loan records from June 2007 to December 2018. There are 36-month and 60-month long loans provided by LCL data. Therefore, there exist quite a few borrowers who belong to the “Current” category out of those who received the loan after 2013. Their default record is unknown. Because of these data problems, we only used loans issued in 2012. In the 2012 loan record, Fully Paid, Default, and Charged Off status existed, and in this study, Fully Paid was defined as a good result and the other two were defined as bad results.

In sum, our dataset consists of 51,314 issued loans, including 8,241 defaults. The LCL dataset describes 145 attributes of borrowers but like previous studies, selected only the important attributes with several references [18, 33, 34]. Brief descriptions of the seven numeric and five categorical attributes used in this study are presented in Table 1. Employment length and home ownership are soft information not directly representing borrowers’ financial status. We removed the missing values for the 12 variables and obtained 37,012 borrowers with fully paid loans and 7,080 borrowers with defaulted loans.

**Table 1:** Description of attributes used in this study.

Type	Variable	Definition
Numeric	Annual income	The annual income provided by the borrower during registration
	Debt to income	The borrower’s debt-to-income ratio: monthly payments on the total debt obligations, excluding mortgage, divided by self-reported monthly income
	Inquiries in the last six months	The number of inquiries by creditors during the past 6 months
	Loan amount	The listed amount of the loan applied for by the borrower
	Open accounts	The number of open credit lines in the borrower’s credit file
	Revolving balance	The total credit revolving balance
	Revolving utilization rate	The amount of credit the borrower is using relative to all available revolving credit
Categorical	Employment length	Employment length in years: integers between 0 and 10, with 0 meaning less than one year and 10 meaning ten or more years
	Grade	Lending Club categorizes borrowers into seven different loan grades from A down to G, A-grade being the safest.
	Home ownership	The home ownership status information provided by the borrower during registration: rent, own, and mortgage
	Loan length	The length of time (years) that workers have been with their current employer: 36 months, 60 months
	Loan purpose	Includes 14 loan purposes: wedding, credit card, car loan, major purchase, home improvement, debt consolidation, house, vacation, medical, moving, renewable energy, educational, small business, and other

**Fig 1:**
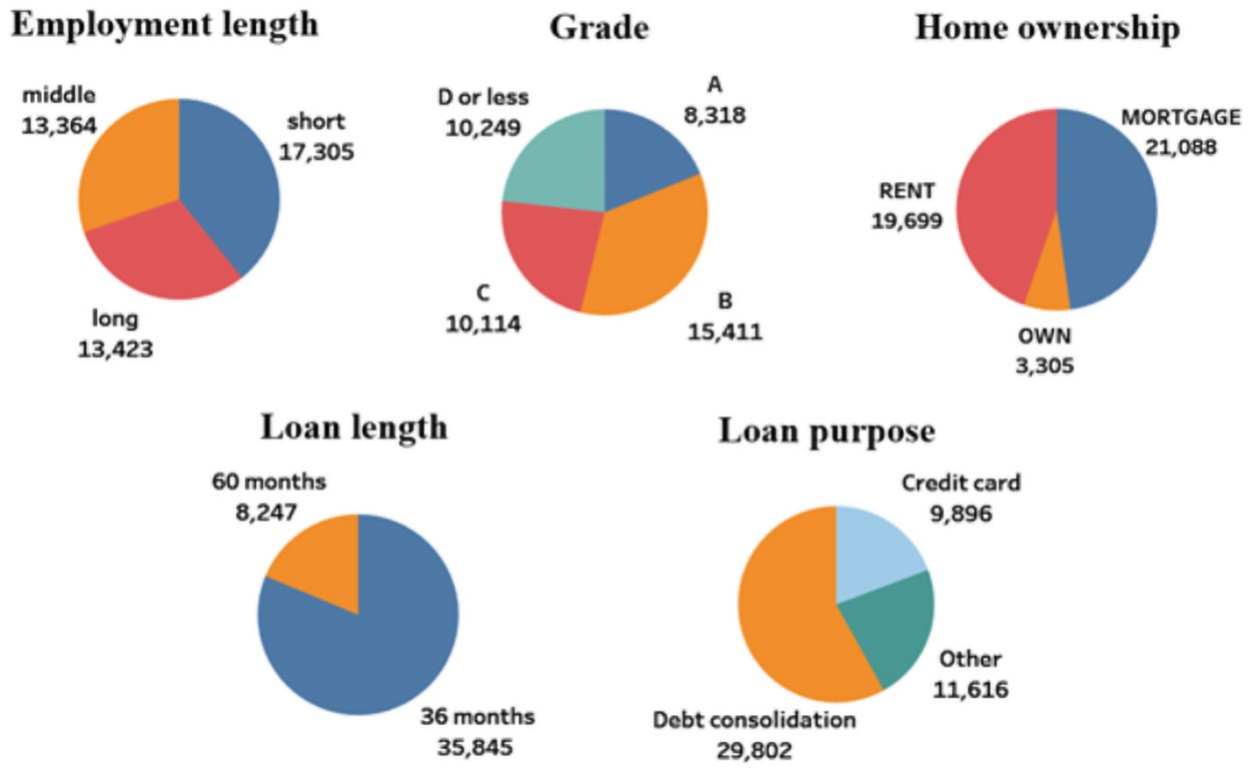
The distribution of categories for each categorical attribute.

We performed preprocessing, taking into account the dispersion of each attribute. “Annual income,” “Loan amount,” and “Revolving balance” are log-transformed to reduce variance. Since 77% of all borrowers are classified as A, B, or C in the "Grade" attribute, classifications D to G are combined together as D or less. Since 78% of all borrowers are also concentrated under the categories debt consolidation and credit card in the "Loan purpose" attribute, we combined the remaining categories into the category other. The "Employment length" attribute is newly categorized as short, representing less than five years; middle, five to nine years; and long, 10 years or more. Thus, the categorical variables increased to nine, and their distribution is shown in Fig 1.

We performed the Welch`s T test on the difference between borrowers with fully paid loans and borrowers with defaulted loans for numeric attributes, as shown in Table 2. There were no statistically significant differences in the "Revolving balance" attribute under the significance level of 0.05. However, for attributes related to income, borrowers with fully paid loans are observed to be more stable than borrowers with defaulted loans.

**Table 2:** Result of the Welch`s T test for numeric attributes.

Attributes	Fully paid loans	Defaulted loans	P-value
Annual income (log)	11.0397	10.9587	<0.0001
Debt to income	16.7235	18.2408	<0.0001
Inquiries in the last six months	0.7908	0.9697	<0.0001
Loan amount (log)	9.2938	9.4174	<0.0001
Open accounts	11.1021	10.757	<0.0001
Revolving balance (log)	9.2420	9.2568	0.29
Revolving utilization rate	57.4090	62.2409	<0.0001

We performed a chi-square test to check if being in default in a categorical attribute is independent of its categories. Table 3 shows for each category the number of borrowers with fully paid loans and those with defaulted loans, the ratio of borrowers with defaulted loans to borrowers with fully paid loans, and the chi-square statistic with the corresponding p-value. Depending on the “Grade” and the “Loan length,” the default-to-fully-paid ratio was quite different. The “Employment length” did not show a statistically significant difference under the p-value of 0.05.

**Table 3:** Result of the chi-squared test for categorical attributes.

Attribute	Category	Fully Paid Loans	Defaulted Loans	Defaulted / Fully Paid Loans	Chi-squared test
Employment length	Short	14,592	2,713	0.19	4.5902 (0.1)
	Middle	11,148	2,216	0.2	
	Long	11,272	2,151	0.19	
Grade	A	7,757	561	0.07	1589.9 (<0.0001)
	B	13,493	1,918	0.14	
	C	8,251	1,863	0.23	
	D or less	7,511	2,738	0.36	
Home ownership	Mortgage	17,935	3,153	0.18	37.839 (<0.0001)
	Own	2,762	543	0.2	
	Rent	16,315	3,384	0.21	
Loan length	36 months	31,030	4,815	0.16	978.29 (<0.0001)
	60 months	5,982	2,265	0.38	
Loan purpose	Credit card	7,365	1,074	0.15	99.942 (<0.0001)
	Debt consolidation	21,432	4,481	0.21	
	Other	8,215	1,525	0.19	

## 5. Experiment

In our dataset, borrowers with defaulted loans account for 16% of the total; thus, there is a class imbalance problem. This leads to a problem whereby the classification model is trained to be biased to predict a major class, and significantly reduces the performance of the prediction of a minor class [35]. To alleviate this problem, we utilized the under-sampling method [36]. We sampled 5,000 borrowers with fully paid loans and 5,000 borrowers with defaulted loans. We limited the range of some numeric attributes to control the dispersion of their min-max normalization. Values greater than 3 for "Inquiries in the last 6 months" and 26 for "Open accounts" were excluded from the sampling process. The spatial weight matrix, W, has been built from the sampled dataset, as described in section 3.2. Numeric variables were divided into three sections of equal length (**L**). The bandwidth (**d**) was set to 0.06059, which was the third quantile value of distances between borrowers.

**Fig 2:**
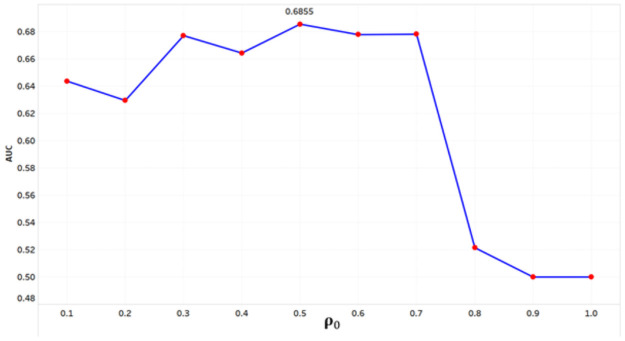
Test AUC variation with initial ρ_0_.

To consider the allowable computation time for parameter estimation, we sampled 2,000 borrowers from the sample dataset, which was divided into 1,500 train datasets and 500 test datasets. Using the train dataset, the parameters: $\hat{\theta }=[\hat{\rho },\hat{\beta }]$ were estimated by GMM. To find the initial **ρ**_**0**_, we observed a change in the “area under the curve” (AUC) for the test dataset by increasing the **ρ**_**0**_ from 0 to 1 at intervals of 0.1. As shown in Fig 2, with an initial **ρ**_**0**_ of 0.5, the test AUC was the highest, at 0.6855. This shows that borrowers are not independent in the borrowers’ relation network, and that there is sufficient spatial autocorrelation between borrowers with defaulted loans.

Table 4 compares the baseline model, logistic regression model without spatial component, with the model presented in this study. In the baseline model, ten attributes were statistically significant at the significance level of 0.1. The default probability of the borrower has a strong negative correlation with the “log(Annual income)” and “log(Revolving balance)” attributes. However, it has a positive correlation with the “Debt to income,” “Revolving utilization rate,” “Grade,” “Loan length,” and “Loan purpose.” In the spatial probit model proposed in this study, seven attributes were statistically significant at the significance level of 0.1. The “log(Annual income)” and “log(Revolving balance)” attributes were underestimated over the baseline model and were not statistically significant. Instead, “log(Loan amount)” and “Revolving utilization rate” have negative coefficients. In addition, the spatial autocorrelation component between borrowers with defaulted loans was 0.505, which was very significant under the significance level of 0.05. Compared to the baseline model, there was an increase in accuracy and AUC. In particular, the proposed model has remarkably increased recall and F1-score, which can be expected to have significant spatial autocorrelation between borrowers with defaulted loans. The additional consideration of spatial autocorrelation in the borrower relation network significantly improved the performance of logistic regression.

**Table 4:** Result of the estimation of the baseline and SAR models.

	Baseline model			Spatial probit model			
	Estimate	Std. Error	Pr(>|Z|)	Estimate	Std. Error		Pr(>|Z|)
Intercept	-0.076	0.563	0.893	-0.481		0.867	0.579
log(Annual income)	-1.714 ***	0.546	0.002	-0.417		0.559	0.455
Debt to income	0.512 *	0.301	0.089	0.963 ***		0.312	0.002
Inquiries in the last 6 months	0.192	0.176	0.276	-0.007		0.180	0.969
log(Loan amount)	0.584	0.384	0.128	-0.905 **		0.404	0.025
Open accounts	0.444	0.361	0.219	-0.557		0.371	0.133
log(Revolving balance)	-2.106 ***	0.786	0.007	0.368		0.813	0.651
Revolving utilization rate	0.591 *	0.327	0.071	-0.766 **		0.334	0.022
Employment length (short)	-0.006	0.131	0.963	-0.039		0.131	0.768
Employment length (long)	0.155	0.142	0.275	0.104		0.143	0.469
Grade (B)	0.457 **	0.194	0.018	0.677 **		0.328	0.038
Grade (C)	0.805 ***	0.213	<0.001	1.085 ***		0.362	0.003
Grade (D or less)	1.081 ***	0.235	<0.001	1.394 ***		0.393	<0.001
Home ownership (Own)	-0.062	0.213	0.771	-0.179		0.213	0.401
Home ownership (Rent)	0.111	0.124	0.391	0.094		0.124	0.446
Loan length (60 months)	0.581 ***	0.163	<0.001	0.488 ***		0.182	0.007
Loan purpose (debt consolidation)	0.269 *	0.155	0.083	0.129		0.154	0.404
Loan purpose (other)	0.395 **	0.189	0.036	0.118		0.187	0.529
Spatial component (**ρ**)				Estimate		*LM*_*ρ*_	p-value
				0.505 ***		273.282	<0.001
Accuracy	0.624			0.652			
Precision	0.63			0.619			
Recall	0.6			0.792			
F1 score	0.615			0.695			
AUC	0.696			0.713			

*, **, and *** represent significance at the 10%, 5%, and 1% levels, respectively.

We sampled the training and test dataset 500 times and observed changes in the test performance differences of the baseline and spatial probit models in the entire dataset. To observe the strength of autocorrelation between borrowers with defaulted loans, the initial **ρ**_**0**_ was set to 0.2, 0.5, and 0.8. The results are shown in Table 5. The larger the initial rho, the higher the recall, which means the higher the predictability of the borrowers with defaulted loans. However, too large an initial value creates the risk of reduced accuracy and AUC. In our experiment, when the initial rho is 0.5, the AUC is slightly higher, and the F1-score is significantly higher than the baseline model. Therefore, a consideration of the appropriate level of spatial autocorrelation is expected to contribute significantly to the prediction of the default risk of a borrower.

**Table 5:** Result of the estimation of the SAR model with 500 repetitions.

Initial Rho	0.2	0.5	0.8
	Mean	Mean	Mean
Accuracy (Baseline model: 0.614)	0.606	0.613	0.592
Precision (Baseline model: 0.612)	0.598	0.590	0.564
Recall (Baseline model: 0.622)	0.647	0.745	0.809
F1 score (Baseline model: 0.617)	0.621	0.658	0.664
AUC (Baseline model: 0.660)	0.650	0.665	0.652

## 6. Conclusion

This study proposed a spatial probit model to improve default prediction by reflecting the relationship between borrowers, which is defined by the similarity of their characteristics.

We applied this method to 2012 LCL data. We found an evidence of a high level of spatial autocorrelation between borrowers with defaulted loans. Reflecting the spatial autocorrelation among loan applicants did not result in an overall improvement in the accuracy of the default prediction but instead, a significant improvement in the F1-score. An increase in the F1 score is a very significant contribution, since finding borrowers with high default risk is a more important issue than finding normal borrower. This study showed that the additional information of spatial autocorrelation between borrowers with high default risk can alleviate the class imbalance problem in the loan dataset and provide a high predictive power for high default risk borrowers.

However, this study has some limitations. Since the spatial weighting matrix increases enormously in proportion to the square of the number of observations, there are time and memory difficulties in using all the data. In addition, the calculation of the inverse of (**I** − **ρW**) in the parameter estimation process using GMM requires a large amount of computation. Because of these constraints on the spatial weighting matrix, we sampled a small number instead of the entire dataset. If the computing power is complemented and the constraints on the spatial weighting matrix are relaxed, then more robust default predictive modeling can be expected.

## Supporting information

S1 File (ZIP)
